# The behavioural effect of short-term cognitive and physical intervention therapies in old dogs

**DOI:** 10.1007/s11357-024-01122-2

**Published:** 2024-04-03

**Authors:** Zsófia Bognár, Dóra Szabó, Borbála Turcsán, Enikő Kubinyi

**Affiliations:** 1 MTA-ELTE Lendület Momentum Companion Animal Research Group, Budapest, 1117 Hungary; 2 Department of Ethology, ELTE Eötvös Loránd University, Budapest, 1117 Hungary; 3 Senior Family Dog Project, Budapest, 1117 Hungary; 4 ELTE NAP Canine Brain Research Group, Budapest, 1117 Hungary

**Keywords:** Dog aging, Age-related decline, Cognitive intervention, Physical intervention, Combined intervention

## Abstract

Efforts to counteract age-related decline have resulted in the emergence of various interventions. However, everyday benefits are rarely reported in elderly people. Dogs provide an excellent model for studying aging and interventions due to their similarities to humans. Our aim was to investigate whether a combined physical and cognitive intervention (most effective in humans) could enhance the performance of pet dogs and lead to far transfer effects (improvement in not just the trained specific task). We examined the impact of three-month-long intervention therapies (cognitive, physical, combined) on the cognitive performance and behaviour of old, healthy dogs (N = 72; aged 7.68–14.54 years) using a 12-subtest behavioural test battery. We did not find the combined intervention group outperforming either the cognitive-only or physical-only therapy groups. Physical interventions, either alone or in combination, improved dogs' behavioural flexibility and social behaviour. Cognitive interventions, either alone or in combination, increased neophilia. Furthermore, all intervention therapies made dogs more engaged with their environment. Moreover, less old, around eight years old dogs, exhibited improved social behaviour, problem solving ability, and increased neophilia by their second test occasion. Additionally, dogs' performance was influenced by their health, training, daily play with the owner, and activity/excitability traits. In sum, both cognitive and physical intervention therapies can have an impact on the behaviour of old, healthy pet dogs. However, these therapies may be more effective when longer or applied at a younger age, as the healthy older dogs were less likely to show improvement.

**Supplementary Information:**

The online version contains supplementary material available at 10.1007/s11357-024-01122-2.

## Introduction

Population aging is a global trend [1] and one of today's biggest challenges. Aging is associated with a decline in the ability to maintain homeostasis [2]. However, the body and the brain are able to adapt to changing conditions in a plastic way after sufficient practice [3–5]. There are many studies aiming to reduce or reverse the cognitive decline associated with aging and, in this context, to develop so-called intervention therapies.

Interventions can be broadly categorised into two types: non-behavioural (pharmaceutical or dietary) and behavioural. Behavioural intervention approaches can be categorised into three subtypes: cognitive, physical, and social interventions [6]. Social isolation has a negative impact on the cognitive health of elderly people, and this connection may also be attributed to the lack of cognitive stimulation [7, 8]. Consequently, social interventions aimed at reducing social isolation can also have advantageous effects on the cognitive function of older individuals. The combination of both physical and cognitive intervention therapies has been put forward as the most promising path. This explanation is thought to be the 'guided plasticity facilitation' hypothesis; that is, physical intervention therapy may 'facilitate' plasticity, while cognitive intervention therapy may 'guide' the changes in plasticity [9]. Consequently, the combination of both types can have synergistic effects. However, it is debated whether cognitive interventions are effective in improving the general day-to-day functioning of old individuals, as 'far transfer effects' (i.e., improvement not only in the specific task they practised during the intervention) are rare and small in effect size [3]. While process-based trainings are more promising when compared to strategy-based trainings, as they target more general cognitive abilities (e.g., working memory, cognitive control) rather than introducing explicit strategies to solve specific tasks, far transfer effects are still rarely reported in older adults [3].

Dogs serve as an excellent model for cognitive aging and intervention studies due to their 1) similarities to humans (environment, age-related changes and diseases); 2) faster aging process (which facilitates easier observation of age-related changes); and 3) rich genetic, phenotypic, and environmental variety, making them ideal for evaluating the effectiveness of aging interventions [10]. Most previous dog studies evaluating cognitive performance in aging dogs in response to interventions, concentrated on dietary interventions in laboratory dogs [11–15] and family dogs [16], while behavioural-only interventions were rare.

The impact of behavioural enrichment on laboratory dogs was also typically studied in conjunction with dietary interventions. These studies consistently reported that behavioural enrichment can slow age-dependent cognitive decline. Furthermore, a synergistic effect was observed when combining long-term behavioural enrichment with an antioxidant diet [17–21]. However, a later study failed to reproduce this effect [22]. In these works, behavioural enrichment included not only cognitive but also physical and environmental intervention. As a result, it is not possible to distinguish the importance of each intervention, but the authors suspected that the cognitive type held particular significance. In humans, both physical and mental activities were reported to have a positive impact on age-related cognitive decline, but combined interventions were reported to be the most effective [23–25]. The question of social interventions is less relevant in the case of pet dogs, as they are less likely to experience social isolation compared to older people. However, social enrichment, that is, housing animals in pairs (as part of the behavioural enrichment), has also been found to have a positive effect on the cognitive performance of laboratory beagles [13, 17, 19, 26].

Although the effects of intervention therapies were mostly studied on laboratory dogs, the effects of life-long activities on cognitive performance were investigated in companion dogs, too. In a questionnaire study, physical activity was found to be associated with better cognitive performance in companion dogs [27]. Life-long cognitive training was also found to have a positive impact on dogs' cognitive performance [28], but other studies failed to find such an association [16, 29].

 To date, there have been no published studies on physical activity-based interventions involving family dogs. Our goal was to test whether physical, cognitive, or combined interventions [23] can lead to detectable improvements in dogs' performance when applied to family dogs, and whether far transfer effects (improved performance on related but untrained tasks or improvement in tests measuring abilities which were not trained directly [3]) are observable.

## Methods

### Ethical statement

The procedures applied in this study complied with national and EU legislation and institutional guidelines, and the study was performed under the recommendations in the International Society for Applied Ethology guidelines for the use of animals in research. A written statement (PE/EA/853–2/2016) was obtained from the local ethical committee (Pest Megyei Kormányhivatal Élelmiszerlánc-Biztonsági és Állategészségügyi Igazgatósága, Budapest, Hungary). According to this statement, the current study is a non-invasive observational experiment; therefore, no special permission is needed according to the corresponding definition by law (‘1998. évi XXVIII. Törvény' 3. §/9. — the Animal Protection Act). The owners of dogs volunteered to take part in the study, and could, at any point, decline to participate. They were informed about the procedures and data handling protocols, and they provided written consent about their dogs' participation. Participants gave written permission to publish their photos.

### Declaration of Generative AI and AI-assisted technologies in the writing process

During the preparation of this work, the authors used Grammarly and ChatGPT 3.5 for grammar correction. After using this tool/service, the authors reviewed and edited the content as needed and take full responsibility for the content of the publication.

### Subjects

Potential subjects (N = 109) were pre-screened before the start of the study, and only healthy animals were enrolled. We defined "healthy" as free from major neurological problems (these were reported by the owners), serious mobility issues, and visual and hearing impairments, which would have prevented them from successfully completing the tasks and detecting stimuli during the tests. Before the experiments, a qualified physiotherapist screened out subjects who suffered from acute or chronic locomotor problems to ensure they would not be harmed by physical intervention therapy. A sensory assessment was also conducted to exclude subjects with possible visual and/or hearing impairments (as suggested by Bognár et al. [30], although the assessment was shorter). We excluded 25 dogs during the sensory/physical assessment due to signs of sensory impairments, signs of conditions affecting mobility, or conditions where participating in this type of regular exercise could risk their condition deteriorating (e.g., luxating patella) or causing pain.

We tested the dogs in a behavioural test battery twice. First, their baseline performance before the intervention therapies and second, after they completed the therapies (or after four months in the case of the control group). The time between the measurements was four months (median ± SD = 4.23 ± 0.76 months). We had to exclude 11 additional dogs because the owner didn't bring back the dog for a second test (due to various reasons, including the death of the dog, the moving of the owner, or the lack of cooperation of the owner). One additional dog had to be excluded because the owner didn't fill out the questionnaire necessary for the analysis.

The final sample included 72 dogs, 52.78% of them were males, and 86.11% were neutered (7 intact males and 3 intact females). The sample consisted of 32 mixed-breed dogs and 40 purebreds from 19 different breeds, their weight ranged from 7 to 80 kg (median weight ± SD = 21.66 ± 11.24 kg). At the time of the baseline measurement, the dogs' age ranged from 7.68 years to 14.54 years (median age ± SD = 10.45 ± 1.63 years).

### Intervention therapies

The dogs were semi-randomly enrolled into four groups: 1) physical therapy (N = 20; median age ± SD = 11.12 ± 1.59 years; 50.00% male); 2) cognitive therapy (N = 19; median age ± SD = 10.27 ± 1.67 years; 57.89% male); 3) combined therapy (N = 24; median age ± SD = 10.49 ± 1.66 years; 50.00% male); and 4) control group (N = 21; median age ± SD = 10.21 ± 1.54 years; 52.38% male). The availability of the owners also determined which group their dogs were assigned to. The therapies lasted for three months (12 occasions), with weekly sessions (1 h long) and homework (see details below). The duration of the intervention therapies was based on [31]. The dogs in the control group did not take part in any therapy sessions, they were just re-tested four months after their baseline assessment. However, a subsample of dogs in the control group (N = 12) was enrolled in one of the therapy groups afterwards. That is, these dogs had three test occasions: 1) baseline measurement, 2) control measurement – intervention therapy baseline, and 3) measurement after the intervention therapy. The rationale for this arrangement was that our call was looking for owners who would be willing to participate in an intervention study. At the same time, a control group was also needed. Therefore, we randomly selected controls from among the applicants, who could only participate in the intervention study later. Since these dogs did not become outliers, we found no reason to exclude them, enabling us to have a larger sample size.

#### Physical intervention therapy

**Fig. 1:**
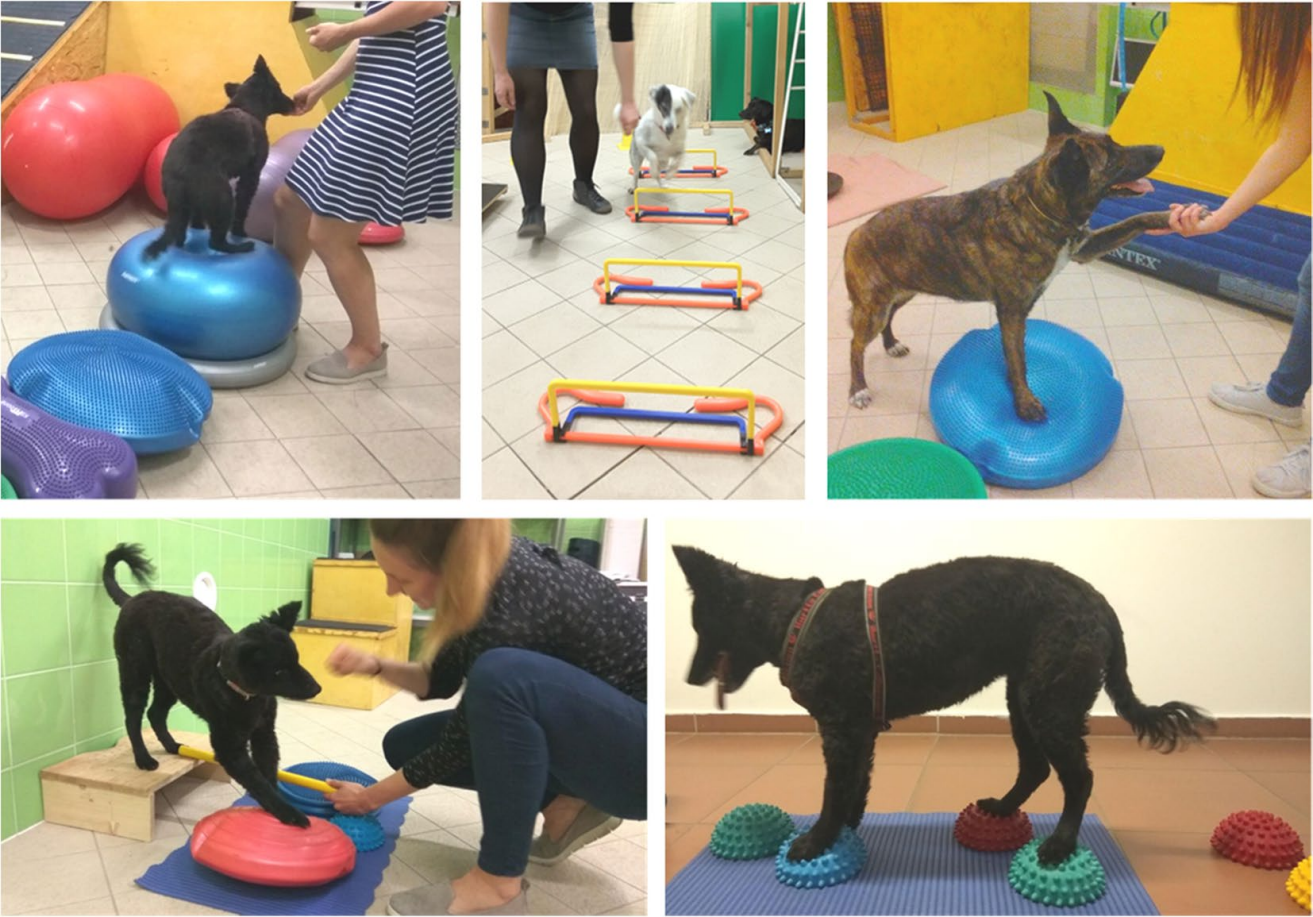
Physical intervention therapy. It focused on balance and stabilization, body awareness, flexibility and endurance using standard fitness equipment and tasks

Physical therapy focused on improving dogs' balance, coordination, joint flexibility, and stamina. The program was based on an age-appropriate fitness program, targeting balance and stabilization, body awareness, flexibility and endurance using standard fitness equipment and tasks (Fig. 1). The goal of these sessions was to strengthen the muscles, promote balanced limb use, improve balance, coordination, and proprioception. Task difficulty and progress were adjusted to the individuals to provide challenging but comfortably doable task difficulties on each occasion. The program was led by certified dog physiotherapists, and on one occasion, the group size was always below five dogs. Among dogs enrolled in this intervention, four dogs previously belonged to the control group.

#### Cognitive intervention therapy

**Fig. 2:**
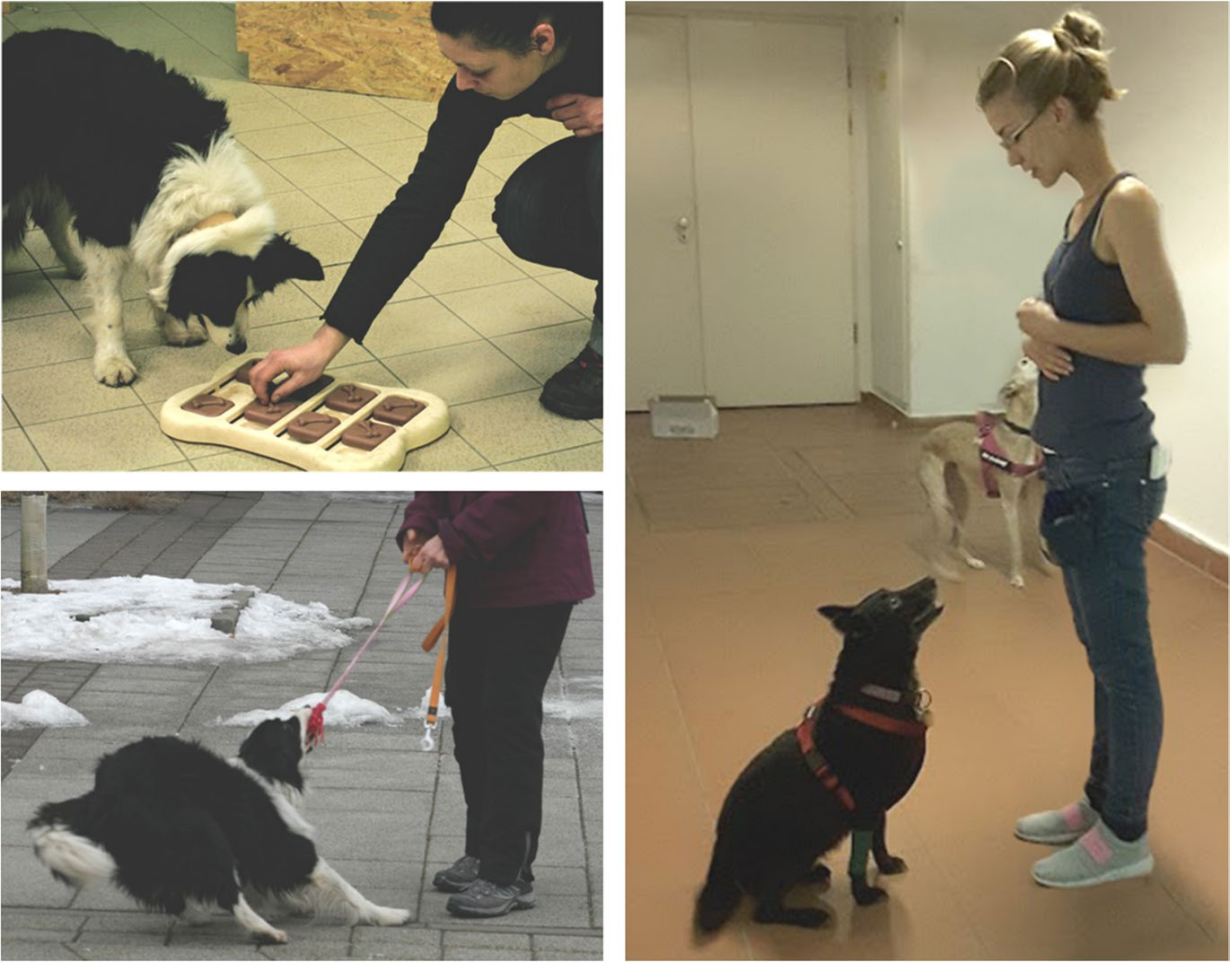
Cognitive intervention therapy. It involved common dog training approaches and areas like clicker training, fetch training, mantrailing, control tasks and interactive dog toys

The cognitive therapy focused on improving dogs' resistance to distraction, processing speed, olfaction, and task switching. The targeted domains were sustained attention, self-control, cognitive flexibility, decreasing reaction time, and problem solving. The exercises involved common dog training approaches and areas like clicker training, fetch training, mantrailing, control tasks and interactive dog toys (Fig. 2). These exercises did not overlap with the test situations. Task difficulty and progress were adjusted to the individuals to provide challenging but comfortably doable task difficulties on each occasion. The program was led by dog trainers and on one occasion, the group size was always below five dogs. Among dogs enrolled in this intervention, two dogs previously belonged to the control group.

#### Combined intervention therapy

Dogs in the combined therapy group took part in both physical and cognitive therapies in weekly alterations (one occasion of physical therapy followed by a cognitive one). Among dogs enrolled in this intervention, six dogs previously belonged to the control group.

### Behavioural test battery

The general aim of the behavioural test battery was to measure a wide range of behaviours and cognitive abilities which are likely to decline with aging. The selection criteria for the tests were as follows: 1) require minimal or no pre-training from socialized family dogs; 2) be repeatable; 3) have as little carry-over effects between subtests as possible; 4) require a minimal amount of equipment (no heavy or large objects which cannot be easily dismounted); 5) be able to be carried out during a total of two visits; 6) only rely on food motivation; and 7) should not exhaust aged dogs. It was also important for us that the tasks resemble the day-to-day functioning of the dogs. Instead of artificial situations, such as training dogs to solve problems on a touchscreen [32] or in a delayed-non-matching-to-sample while the dog is sitting in a box [33], we strove to model tasks after everyday situations. See the list of the 12 tasks in the behavioural test battery in Table 1.

**Table 1:** List of the tasks in the behavioural test battery, their aims and the everyday situations they aim to model

Test	Aim	Everyday situation
*1. Exploration test* [79]	To measure the dog's activity and exploration in a room containing a wide range of objects	The dog is at a new place
*2. Box rustle test* [79]	To measure the dog's interest in and following the owner's activity	The owner moves around the house and searches for something
*3. Greeting test* [57, 80]	To assess the dog's social behaviour/responsiveness toward an unfamiliar human, first during a greeting situation (while the dog was on a leash), then in an object play situation (the dog was off-leash)	The dog meets a new person who tries to interact with the dog
*4. Pointing test* [81]	To measure the dog's ability to follow a momentary human pointing gesture when locating a hidden food reward, and also the dog's ability to shift from a previously rewarded response, by pointing to the same side three times in a row, then to the other side during the following three trials	The owner directs the dog's attention to something by pointing
*5. Manipulative persistency test* [82]	To measure the dog's willingness and ability to obtain treats from an interactive toy and its persistence in trying to obtain an inaccessible food reward	The dog gets a feeding toy to keep them occupied for a while
*6. Clicker game* [83]	To measure the dog's one-trial learning ability to associate a given behaviour with food reward after a single trial, and also behavioural flexibility, that is, its ability and willingness to offer novel behaviours to the experimenter in a positive reinforcement setup	A training situation where the owner teaches the dog something very simple but new
*7. Problem solving test* [84]	To assess the dog's individual problem solving when locating a hidden food reward in a problem box, together with the dog's behavioural inhibition and flexibility, by systematically shifting the visibility of the food reward and the location of the opening on the problem box	The owner hides something that the dog has to find
*8. Attention test* [28, 85]	To measure the dog's attentional capture and sustained attention in a social and non-social context	The dog needs to pay attention to other moving living creatures (e.g., humans, conspecifics) or inanimate objects (e.g., cars, vacuum cleaners)
*9. Training for eye contact test* [28, 57, 83]	To measure the dog's associative learning ability to sustain social attention, that is, to learn the association between establishing eye contact with the experimenter and the food reward, and then sustain eye contact with the experimenter for increasing durations	For communication, cooperation and training, the dog needs to pay attention to their owner, look at them, and keep eye contact
*10. Novel object recognition* [86]	To assess the dog's reaction towards novel and familiar toys and potential preference for any of them	The dog gets a new toy
*11. Memory test* [87]	To measure the dog's short-term visuo-spatial memory	The owner hides something that the dog has to find. Or the dog has to remember where they left something (e.g. a ball)
*12. Discrimination and reversal learning* [88–90]	To assess the dog's ability to learn the association between the colour or the location of a stimulus and the reward, as well as the dog's ability to re-learn the association when the positive and negative stimuli were reversed	The dog needs to learn to associate irrelevant cues with positive or negative stimuli

The experimenters were different on the two measurement occasions and were randomized among the dogs. The entire battery took approx. 120 min to complete and was carried out on two separate days (median ± SD = 9.00 ± 10.66 days difference), with the first eleven tasks (approx. 1 h long) on the first day and the twelfth task (approx. 1 h long) on the second day. The tests were provided in a fixed order for all subjects (similarly to [34, 35]) to ensure consistency across subjects and make direct comparisons of individual differences across tasks and among repeated measurements possible. A video of the protocol can be found on the following link: https://youtu.be/bO80tYgAvJI. The whole behaviour test battery consisted of twelve tasks. The brief aims of these tasks can be found in Table 1. The detailed behavioural test battery protocol can be found in the Supplementary material.

The tasks were video-recorded, and we coded the behaviour using Solomon Coder beta 19.08.02 (copyright 2006–2019 by András Péter) and a scoring sheet. Initially, we coded 100 variables but later excluded 39 variables (for more details, see Supplementary material). The remaining 61 variables (Supplementary Table S1) consisted of 16 nominal scores, 12 durations, 15 frequencies, 17 latencies, and 1 composite score (average of different score-type variables). These variables were used in the subsequent analyses.

To determine the inter-observer reliability, a second observer independently coded a randomly selected sample of 20 dogs for each task. The reliability between observers was calculated using the Intraclass correlation coefficient (ICC) for all raw variables. We found that all observations were reliable (for more details, see Supplementary Table S1).

### Calculating the performance in each subtest

We used principal component analysis (PCA) on the baseline test of a larger pool of subjects (N = 129; see Bognár et al. [36]) to derive composite scores that represent the animal's overall performance in the tasks. The subjects of the current study are a subsample of this previous larger pool. We ran a PCA with Oblimin rotation for each task separately, except for the Pointing test and the Discrimination and reversal learning, where only one and two variables were coded, respectively. Additionally, by extracting components that represent shared variance across multiple variables, we were able to reduce measurement error and other variable-specific effects [37]. In cases where the variables were categorical, the PCA was run based on a polychoric correlation matrix; otherwise, normal correlation matrixes were used. The number of components extracted was decided by parallel analysis in all analyses. Variables that failed to load > 0.5 on any components were removed. We used Kaiser–Meyer–Olkin (KMO) measure and Bartlett's sphericity test to determine the sampling adequacy and Cronbach's alpha coefficient to assess the internal consistency of the items.

We extracted 12 components in total (Supplementary Table S2). The KMO value was > 0.5 for all analyses. In 8 out of 10 task-level analyses, all variables loaded on a single component, suggesting that they all share a common source of variance. In two tasks (Clicker game and Novel object recognition), we found two components. The component scores for both the baseline and the second measurement were calculated manually based on the PCA tables. Data from the two test occasions were pooled together and standardized, and then the component scores were calculated by taking the mean of the standardized (z-score) items loading above 0.5 on a given component. We allowed a maximum of one value to be missing per individual per performance variable, but only if the component was composed of three or more items. In the Pointing test, we coded only one variable, the number of successful trials (out of 6), while in the Discrimination and reversal learning, we analysed the two raw variables (i.e., the N of trials required to learn the initial discrimination and the reversal associations).

Next, we investigated the task reliability over time, using Intraclass correlation (ICC, two-way mixed model). The Exploration, the Box rustle, the Pointing, the Attention, and the Discrimination and Reversal learning tests were found to have low repeatability (ICC < 0.5; see Supplementary Table S2), which could mean that a higher portion of the individual variance in this task is due to random or measurement error or factors unrelated to cognition [38]. Thus, these tests were excluded from further analyses.

All in all, we investigated the effect of intervention therapies on a total of 9 performance variables (9 components derived from the task-level PCAs, see in the Results).

### Calculating the change of performance in each subtest

Since there are intra-individual variations in the dogs' performance in the tests, a slight change between a dog's baseline and second measurements does not necessarily imply a significant positive or negative change. However, we do not have predetermined thresholds, which we could use to differentiate between individual variation (i.e., stable performance) and real change. Therefore, to analyse the effects of interventions on performance, we categorized changes in dogs' performance as objectively as possible.

To objectively categorize these changes, we strove to identify the most appropriate threshold criteria for dividing the data into three groups: [1] declining, [2] stable, and [3] improving. Such criteria ensure balanced distribution, meaning there are no significant differences between the groups in terms of the number of dogs. We found that a 10% change compared to the baseline measurement was the most suitable threshold. Consequently, we defined stable performance as any change of less than 10% between the baseline and second measurements.

If the magnitude of change between the baseline and second measurements exceeded 10% in the negative direction, the dog was categorized into the declining group. Conversely, if the change exceeded 10% in the positive direction, it was classified into the improving group. To eliminate the impact of experience on the change, we calculated the performance change above the median change between baseline and second measurements.

The categorization of performance change for all nine performance variables was calculated as follows:

1. First, we calculated the range of dogs' baseline performance:
  $$range = maximum\;baseline\;performance\;value - minimum\;baseline\;performance\;value$$
2. Then, the value of one percentage change:
  $$percentage = (range)/100$$
3. The tasks' *experience effect* was calculated as the difference between the median value of the second performance value and the median baseline performance value:
  $$experience\;effect = median\;second\;test\;performance\;value - median\;baseline\;performance\;value$$
4. The threshold for performance changes was defined as follows:
  A) The threshold for performance decrease was set at a minimum of a 10% decrease over to the experience effect:
    $$threshold\;for\;performance\;decrease = \left(-10\times percentage\right) + experience\;effect$$
  B) The threshold for performance improvement was set at a minimum of a 10% increase over to the experience effect:
    $$threshold\;for\;performance\;improvement = \left(10\times percentage\right) + experience\;effect$$
5. Then, we calculated the individuals' *performance change* between their second test performance and baseline performance:
  $$change\;value = second\;test\;performance\;value - baseline\;performance\;value$$
  A) If its value was lower than or equal to the value of the negative change's threshold, we categorized the dog as declining:
    $$declining\;IF: change\;value \le threshold\;for\;performance\;decrease$$
  B) If its value was higher than the value of the negative change's threshold and lower than or equal to the value of the positive change's threshold, we categorized the dog as stable:
    $$stable\;IF: threshold\;for\;performance\;decrease < change\;value \le threshold\;for\;performance\;improvement$$
  C) If its value was higher than the value of the positive change's threshold, we categorized the dog as improving:
    $$improving\;IF: threshold\;for\;performance\;improvement < change\;value$$

### Moderator variables

The participating owners also filled in multiple questionnaires, and we used some of these data to investigate if these variables have a moderating effect on the change in the performance or the effect of the intervention on it.

From the demographic questionnaire described in Wallis et al. [39, 40], we used the age of the dog (year), sex (male, female), daily playing activity with the owner (< 1 h, > 1 h), and daily off-leash activity (< 1 h, 1-3 h, > 3 h). Additionally, we created two composite scores from multiple raw variables [36]. The first composite score (health status) was created by averaging the score values of the questions about the necessary regular medication (no/yes), treating the dog with vitamins/supplements [almost never/rarely/often/regularly(daily)] and the number of health problems listed by the owner (no problems/1/2/3 or more). The second composite score (training level) was created by averaging the score values of the questions about the number of commands the dog performs reliably (maximum 3/4/5/6/7 or more), the number of commands known by the dog according to the owner (less than 10/10 or more), number of training types the dog finished (0/1/2 or more), the number of training activities the dog currently attends (0/1/2 or more), the number of leisure activities (1 or less/2/3 or more) and the owner experience score (minimal experience/moderate/professional). From the personality questionnaire (Dog Personality Questionnaire, DPQ [40, 41], we analysed only two traits, Activity/excitability (12 items), and Responsiveness to training (6 items).

### Statistical analysis

For statistical analysis, we used R 4.2.2 software [42]. While a subsample of dogs in the control group (N = 12) subsequently enrolled in one of the therapy groups, the variance explained by the dogs' ID was low for all the nine performance variables (median variance ± SD = 0.00 ± 0.32), indicating that mixed models are not necessary. Therefore, to examine the effect of various factors on the dogs' performance change for all nine performance variables, we used a Multinomial Log-linear Model ("multinom" function of the "nnet" package [43]). We analysed the effect of 1) baseline performance, 2) intervention therapy group, 3) demographic factors (age, sex, daily playing activity with the owner, daily off-leash activity, health status, training level), and 4) personality (Activity/excitability, Responsiveness to training) on each test separately. The effect of the intervention therapy group was examined in four different ways:

1. Comparing the four intervention groups: physical, cognitive, combined and control group.
2. Whether the dog participated in any physical therapy: yes (physical or combined therapy) or no (cognitive therapy or control group).
3. Whether the dog participated in any cognitive therapy: yes (cognitive or combined therapy) or no (physical therapy or control group).
4. Whether the dog participated in any intervention therapy: yes (physical, cognitive, or combined therapy) or no (control group).

To find the most parsimonious models of the performance change for all nine performance variables, bottom-up, AIC-based model selection was used ("anova" function of the "stats" package [42]). The criteria for inclusion in the model were a significant likelihood ratio test for each tested variable and a minimum of two value differences between the compared models. To calculate odds ratios, we used the "OR.multinom" function of the "RVAideMemoire" package [44].

To check the presence of multicollinearity among the independent variables, we used the variance inflation factor (VIF; "vif" function of the "car" package [45]). However, it is important to note that VIF cannot be directly applied to Multinomial Log-linear Models. Therefore, we made Binomial Generalized Linear Models and conducted the VIF analysis on those. This approach provided a close approximation of the potential multicollinearity in our analysis.

## Results

At baseline, there were no significant differences between the intervention therapy groups for any performance components.

According to the model selection, the most parsimonious models on the dogs' performance change for all nine performance components were (for statistical details, see Table 2 and below):

1. Friendliness (*Greeting test*): dogs' baseline performance, participation in any physical therapy, dogs' age, dogs' Activity/excitability score
2. Persistency *(Manipulative persistency test)*: dogs' baseline performance
3. Flexibility (*Clicker game*): dogs' baseline performance, participation in any physical therapy, dogs' training level, dogs' Activity/excitability score
4. One-trial learning (*Clicker game*): dogs' Activity/excitability score
5. Problem solving success (*Problem solving test*): dogs' baseline performance, dogs' age
6. Associative learning (*Training for eye contact*): dogs' baseline performance
7. Preference for novelty (*Novel object recognition*): dogs' baseline performance, participation in any cognitive therapy, dogs' age, dogs' health status, daily playing activity with the owner
8. Preference for familiar (*Novel object recognition*): dogs' baseline performance, participation in any intervention therapy
9. Memory (*Memory test*): dogs' baseline performance

**Table 2:** Summary of the main results, main effects' p-values, and the direction of the association. + : Better performance on the second occasion; o: Stable performance on the second occasion; -: Worse performance on the second occasion

Tests and components		Baseline performance	Intervention therapies	Dog's demographics	Other factors
**Greeting test—Friendliness**		***Higher baseline performance***: *- (p* < *0.001)*	***Participation in any form of physical therapy***: ** + ***(p* = *0.057)*	***Older age***: **-** *(p* = *0.011)*	***More active/excitable dogs***: ** + ***(p* = *0.011)*
**Manipulative persistency test—Persistency**		***Higher baseline performance***: **-** *(p* < *0.001)*	ø	ø	ø
**Clicker game**	**Flexibility**	***Higher baseline performance***: **-** *(p* < *0.001)*	***Participation in any form of physical therapy***: **o** *(p* = *0.049)*	***Higher trained dogs***: **-** *(p* = *0.024)*	***More active/excitable dogs***: ** + ***(p* = *0.067)*
	**One-trial learning**	ø	ø	ø	***More active/excitable dogs***: o *(p* < *0.001)*
**Problem solving test – Problem solving success**		***Higher baseline performance***: **-** *(p* < *0.001)*	ø	***Older age***: **-** *(p* = *0.008)*	ø
**Training for eye contact—Associative learning**		***Higher baseline performance***: **-** *(p* < *0.001)*	ø	ø	ø
**Novel object recognition**	**Preference for novelty**	***Higher baseline performance***: **-** *(p* < *0.001)*	***Participation in any form of cognitive therapy***: + *(p* = *0.029)*	***Older age***: **-** *(p* = *0.008)*	***Healthier dogs***: **-** *(p* = *0.014)*
					***More time spent playing daily***: ** + ***(p* = *0.006)*
	**Preference for familiar**	***Higher baseline performance***: **-** *(p* < *0.001)*	***Participation in any form of intervention therapy***: ** + ***(p* = *0.077)*	ø	ø
**Memory test—Memory**		***Higher baseline performance***: **-** *(p* < *0.001)*	ø	ø	ø

VIF scores were between 1.00 and 2.32, indicating no multicollinearity. The summary of the results can be read in Table 2.

### Greeting test—Friendliness

The general experience effect in the greeting test was lower than 10%.

Dogs who had lower baseline scores were more likely to improve (N = 31) than remain stable (N = 35; ß ± SE: 0.17 ± 0.05; Z = 3.37; OR = 1.19 [1.07—1.31]; p < 0.001) or experience a decline in their performance (N = 18; ß ± SE: 0.23 ± 0.06; Z = 3.62; OR = 1.26 [1.11—1.42]; p < 0.001).

**Fig. 3:**
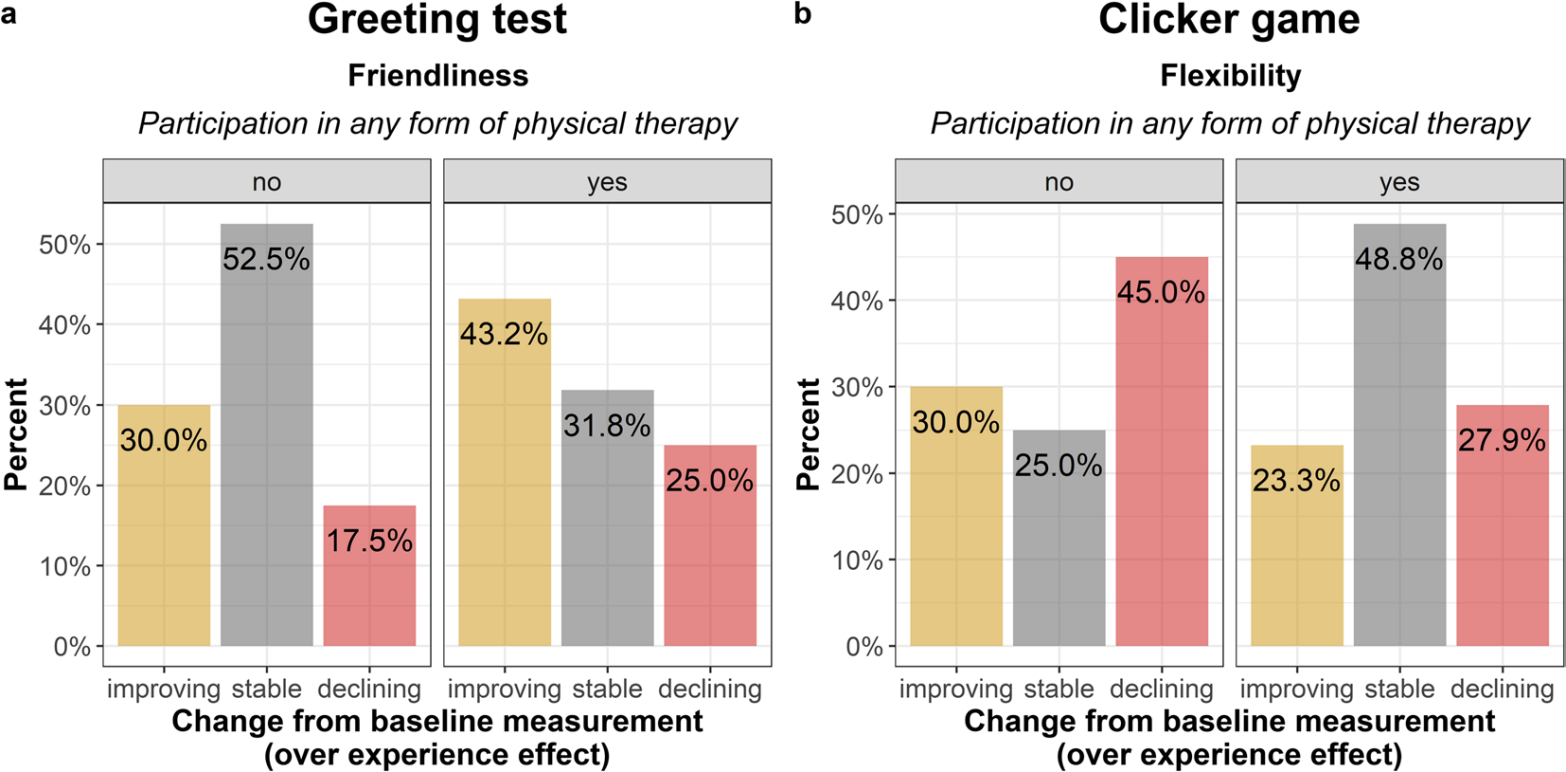
Effect of any form of physical therapy on dogs' social behaviour (**a**) and behaviour flexibility (**b**). Dogs exhibited increased friendliness towards a stranger during the Greeting test. In the Clicker game, performance remained stable for dogs that received physical therapy, while dogs without therapy showed a decline in performance

Dogs who participated in any physical therapy were more likely to improve than remain stable (ß ± SE: 1.10 ± 0.64; Z = 2.03; OR = 3.47 [1.04—11.50]; p = 0.042; Fig. 3A) compared to those who did not participate.

Younger dogs were more likely to improve than remain stable (ß ± SE: 0.54 ± 0.21; Z = 2.58; OR = 1.71 [1.14—2.57]; p = 0.010) or experience a decline in their performance (ß ± SE: 0.55 ± 0.23; Z = 2.37; OR = 1.74 [1.10—2.76]; p = 0.018).

Dogs with higher Activity/excitability scores were more likely to improve than remain stable (ß ± SE: 0.18 ± 0.07; Z = 2.74; OR = 1.20 [1.05—1.37]; p = 0.006).

### Manipulative persistency test—Persistency

The general experience effect in the manipulative persistency test was higher than 10% and negative.

Dogs who had lower baseline scores were more likely to improve (N = 34) than remain stable (N = 29; ß ± SE: 0.07 ± 0.03; Z = 2.41; OR = 1.08 [1.01—1.14]; p = 0.016) or experience a decline in their performance (N = 19; ß ± SE: 0.15 ± 0.04; Z = 3.76; OR = 1.16 [1.07—1.26]; p < 0.001). Also, dogs who had lower baseline scores were more likely to remain stable than decline (ß ± SE: 0.09 ± 0.04; Z = 2.07; OR = 1.08 [1.00—1.16]; p = 0.039).

### Clicker game

The general experience effect in the clicker game (both components) was higher than 10% and positive.

#### Flexibility

Dogs who had lower baseline scores were more likely to improve (N = 22) than remain stable (N = 31; ß ± SE: 0.20 ± 0.08; Z = 2.47; OR = 1.22 [1.04—1.44]; p = 0.013) or experience a decline in their performance (N = 30; ß ± SE: 0.53 ± 0.12; Z = 4.53; OR = 1.69 [1.35—2.13]; p < 0.001). Also, dogs who had lower baseline scores were more likely to remain stable than decline (ß ± SE: 0.32 ± 0.09; Z = 3.50; OR = 1.15 [1.30—1.66]; p < 0.001).

Dogs who participated in any physical therapy were more likely to remain stable than experience a decline in their performance (ß ± SE: 1.60 ± 0.68; Z = 2.35; OR = 4.94 [1.30—18.72]; p = 0.019; Fig. 3B) compared to those who did not participate.

Dogs who had lower training levels were more likely to improve than experience a decline in their performance (ß ± SE: 0.20 ± 0.09; Z = 2.27; OR = 1.22 [1.03—1.44]; p = 0.023), as well as they were more likely to remain stable than decline (ß ± SE: 0.17 ± 0.07; Z = 2.29; OR = 1.18 [1.02—1.36]; p = 0.022).

Dogs who had higher Activity/excitability scores were more likely to improve than remain stable in their performance (ß ± SE: 0.15 ± 0.07; Z = 2.19; OR = 1.16 [1.02—1.33]; p = 0.028).

#### One-trial learning

Dogs who had higher Activity/excitability scores tended to rather remain stable (N = 21) than change (improve (N = 25): ß ± SE: 0.12 ± 0.07; Z = 1.82; OR = 1.13 [0.99—1.29]; p = 0.069; decline (N = 37): ß ± SE: 0.22 ± 0.07; Z = 3.28; OR = 1.25 [1.09—1.42]; p = 0.001). Also, dogs who had higher Activity/excitability scores tended to improve rather than experience a decline in their performance (ß ± SE: 0.10 ± 0.05; Z = 1.80; OR = 1.10 [0.99—1.23]; p = 0.072).

### Problem solving test—Problem-solving success

The general experience effect in the problem solving test was lower than 10%.

Dogs who had lower baseline scores were more likely to improve (N = 16) than remain stable (N = 58; ß ± SE: 0.39 ± 0.11; Z = 3.57; OR = 1.48 [1.19—1.84]; p < 0.001) or experience a decline in their performance (N = 9; ß ± SE: 0.25 ± 0.11; Z = 2.26; OR = 1.29 [1.03—1.60]; p = 0.024).

Younger dogs were more likely to improve than experience a decline in their performance (ß ± SE: 0.66 ± 0.28; Z = 2.50; OR = 3.17 [1.28—7.81]; p = 0.012), as well as more likely to remain stable than decline (ß ± SE: 1.15 ± 0.46; Z = 2.32; OR = 1.93 [1.11—3.37]; p = 0.020).

### Training for eye contact—Associative learning

The general experience effect in the training for eye contact was lower than 10%.

Dogs who had lower baseline scores were more likely to improve (N = 17) than remain stable (N = 50; ß ± SE: 0.13 ± 0.03; Z = 3.77; OR = 1.14 [1.06—1.22]; p < 0.001) or experience a decline in their performance (N = 15; ß ± SE: 0.11 ± 0.04; Z = 2.60; OR = 1.11 [1.03—1.21]; p = 0.009).

### Novel object recognition

The general experience effect in the novel object recognition (both components) was lower than 10%.

#### Preference for novelty

Dogs who had lower baseline scores were more likely to improve (N = 31) than experience a decline in their performance (N = 22; ß ± SE: 0.27 ± 0.08; Z = 3.51; OR = 1.31 [1.13—1.53]; p < 0.001), as well as they were more likely to remain stable than decline (N = 29; ß ± SE: 0.28 ± 0.08; Z = 3.59; OR = 1.32 [1.14—1.54]; p < 0.001).

**Fig. 4:**
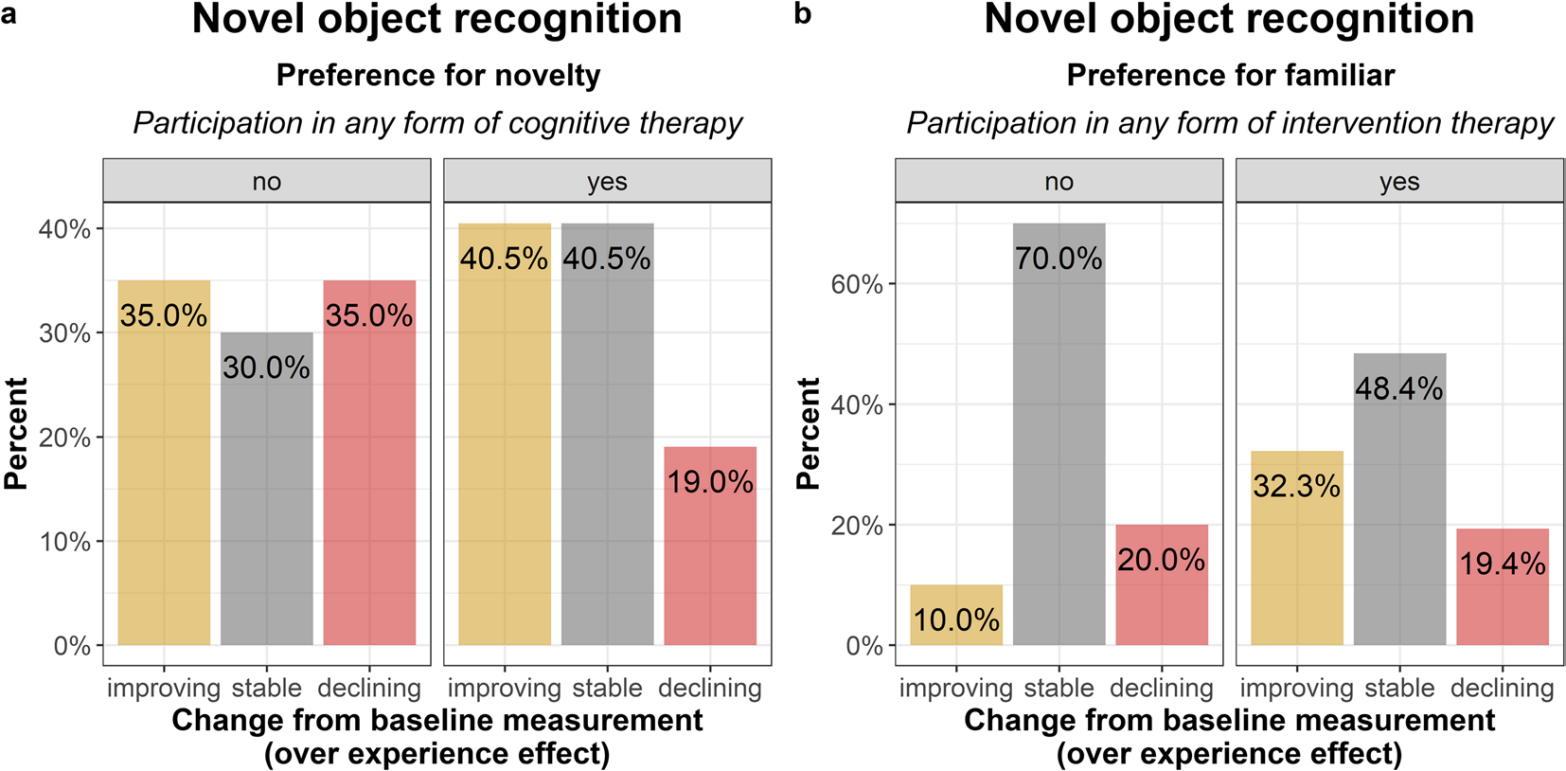
Effect of intervention therapy on dogs' behaviour in the Novel object recognition. Dogs that did not participate in any form of cognitive therapy exhibited fewer manipulations of the new object (**a**). Participation in any form of intervention therapy had a positive impact on the manipulation of the familiar object (**b**)

Dogs who participated in any cognitive therapy tended to rather improve than experience a decline in their performance (ß ± SE: 1.70 ± 0.88; Z = 1.94; OR = 5.47 [0.98—30.49]; p = 0.052) and were more likely to remain stable than decline (ß ± SE: 2.16 ± 0.89; Z = 2.43; OR = 8.70 [1.52—49.64]; p = 0.015; Fig. 4A) to those who did not participate.

Younger dogs were more likely to improve than remain stable (ß ± SE: 0.55 ± 0.21; Z = 2.67; OR = 1.74 [1.16—2.62]; p = 0.008) or experience a decline in their performance (ß ± SE: 0.55 ± 0.26; Z = 2.09; OR = 1.73 [1.04—2.90]; p = 0.037).

Dogs with more health problems were more likely to remain stable than to experience a decline in their performance (ß ± SE: 0.19 ± 0.08; Z = 2.55; OR = 1.21 [1.05—1.40]; p = 0.011).

Dogs that played for more than an hour with their owner on a daily basis were more likely to improve than experience a decline in their performance (ß ± SE: 2.40 ± 0.87; Z = 2.76; OR = 11.01 [2.00—60.45]; p = 0.006), as well as they were more likely to remain stable than decline (ß ± SE: 2.32 ± 0.87; Z = 2.67; OR = 10.19 [1.85—56.09]; p = 0.008) compared to those who played for less than an hour.

#### Preference for familiar

Dogs who had lower baseline scores were more likely to improve (N = 22) than experience a decline in their performance (N = 16; ß ± SE: 0.18 ± 0.05; Z = 3.40; OR = 1.20 [1.08—1.34]; p < 0.001), as well as they were more likely to remain stable than decline (N = 44; ß ± SE: 0.21 ± 0.05; Z = 3.99; OR = 1.23 [1.11—1.36]; p < 0.001).

Dogs who participated in any intervention therapy tended to rather improve than remain stable (ß ± SE: 1.52 ± 0.81; Z = 1.88; OR = 4.59 [0.94—22.48]; p = 0.060; Fig. 4B) compared to those who did not participate.

### Memory test—Memory

The general experience effect in the memory test was lower than 10%.

Dogs who had lower baseline scores were more likely to improve (N = 21) than remain stable (N = 39; ß ± SE: 0.23 ± 0.06; Z = 3.60; OR = 1.25 [1.11—1.42]; p < 0.001) or experience a decline in their performance (N = 22; ß ± SE: 0.18 ± 0.06; Z = 2.80; OR = 1.19 [1.05—1.35]; p = 0.005).

**Fig. 5:**
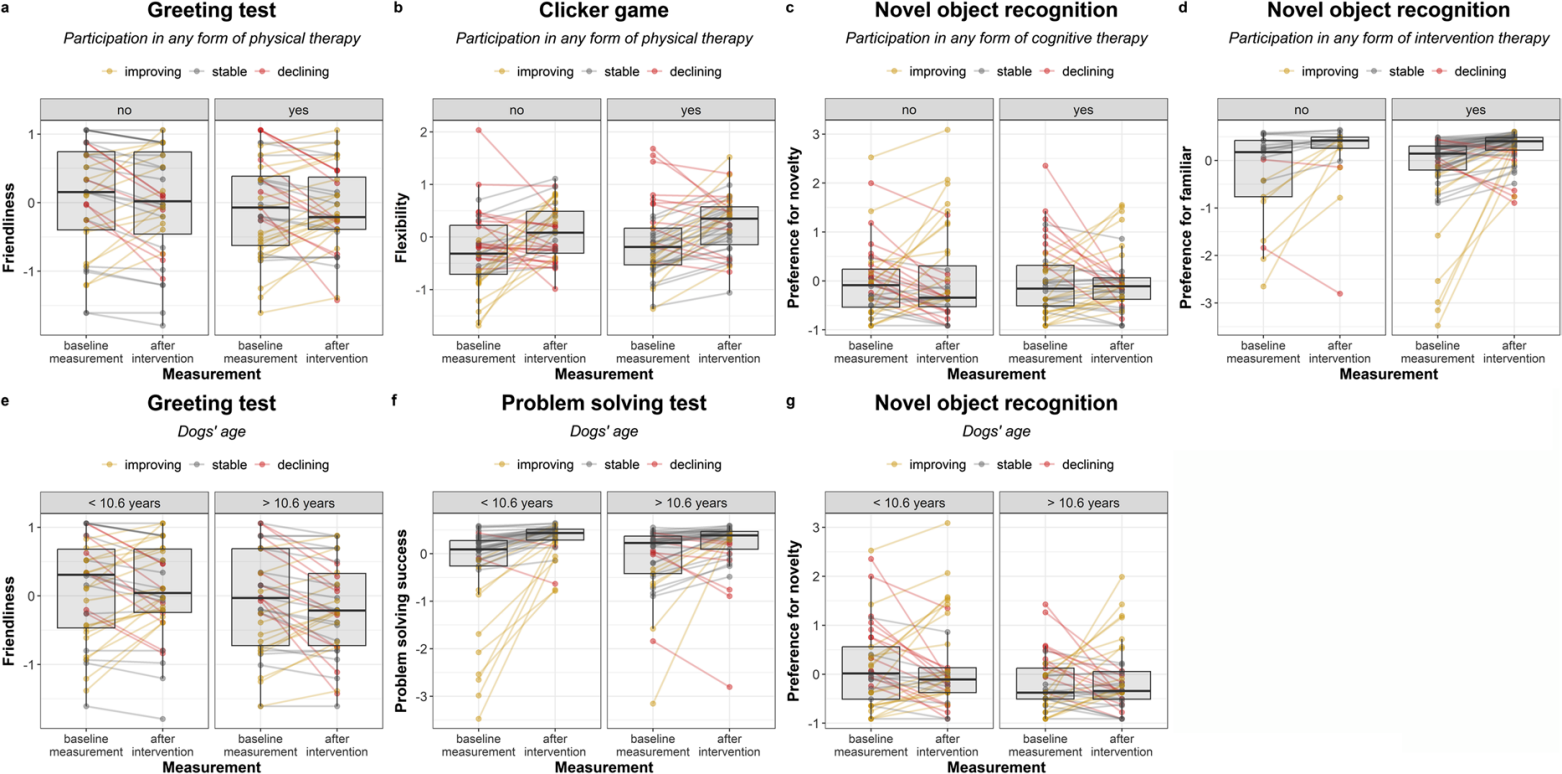
Effects of intervention therapy** (a-d**) and age** (e–g**) on dogs' behaviour. Participation in any form of physical therapy influenced dogs' performance in the Greeting test (**a**) and the Clicker game (**b**). In the Novel object recognition, participation in any form of cognitive therapy influenced dogs' preference for novelty (**c**), while participation in any form of intervention therapy influenced their preference for the familiar object (**d**). Age influenced dogs' performance in the Greeting test (**e**), the Problem solving test (**f**), and the Novel object recognition (**g**). In these three graphs, the sample was divided at the median age (10.6 years) for visualization

The main findings, including the effects of intervention therapy and age, as well as the raw performance scores per behaviour tasks, both at the baseline measurement and the measurement after intervention, are depicted in Fig. 5.

## Discussion

We investigated whether intervention therapy could enhance the performance of old, healthy pet dogs while considering other moderating factors. Additionally, we examined whether these interventions could lead to 'far transfer effects', meaning whether dogs would improve in the untrained tasks. Since we studied three intervention therapy groups (cognitive-only, physical-only, and combined), we were able to assess the importance of each intervention type. We observed that both cognitive and physical therapies improved certain abilities, and we also found other moderating factors to have an impact on the performance change. Since we strove to ensure that the chosen tests resemble everyday situations instead of artificial ones, these results suggest that the intervention therapies also improved the day-to-day functioning of old dogs.

### Effect of intervention therapies

Dogs that participated in any form of physical therapy, both in the physical therapy-only or combined therapy groups, became more sociable and friendlier with the stranger during the Greeting test by their second test occasion. This suggests that regular positive experiences with various humans in physical intervention trainings boosted the dogs' sociability. Furthermore, their performance during the Clicker game was more stable, while dogs without physical therapy rather declined. Given the generally positive experience effect observed in this task (i.e., the median performance improvement between the baseline and second measurement was higher than 10%), the performance of dogs that did not participate in any form of physical therapy (control or cognitive therapy groups) did not necessarily decline compared to the baseline performance, but it could also improve. Nevertheless, this improvement did not reach the extent of the experience effect. This seems to be a natural change with aging, while during physical training, dogs maintained their propensity to offer different behaviours for food rewards, as physical interventions mainly involved such activities. Dogs had to offer new behaviours using dog fitness devices weekly in exchange for food.

Physical activity has emerged as an important protector against cognitive decline both in humans and animal models (e.g., [46, 47]). Since companion dogs may spontaneously develop age-associated Alzheimer-like pathology [48], they serve as the best models for investigating associations between physical activity and cognitive dysfunction, especially Alzheimer's disease. Engaging in physical activity throughout their lives was linked to lower scores on a cognitive dysfunction scale in companion dogs [27]. Future studies should investigate whether physical interventions influence the structure of brain tissue, similarly to how they do in humans [49].

The performance of dogs that participated in any form of cognitive therapy, both in the cognitive therapy-only or combined therapy groups, was more stable during the Novel object recognition, and it improved rather than declined in terms of the manipulation of the new object. Cognitive interventions focused more on improving attention to detail, processing and recalling information, which could impact the recall aspect of the Novel object recognition, making dogs more inclined to seek novelty. Meanwhile, participation in any form of intervention therapy, whether in the physical therapy-only, cognitive therapy-only or combined therapy groups, positively affected the manipulation of the familiar object during the dogs' second test occasion. All intervention types were designed to stimulate the dogs, making them more engaged with their environment. Thus, manipulating the familiar toy in the Novel object recognition can be seen as showing more interest in their surroundings and being more playful. Daily play activities with the owner also influenced their performance change in this task, but only with the novel toy.

Previous studies have suggested that combining physical and mental activities can have a greater effect on cognitive performance than either alone [23]. This is attributed to the 'guided plasticity facilitation' effect, where physical intervention therapy is thought to 'facilitate plasticity,' while cognitive intervention therapy 'guides' the changes [9]. However, we did not find the combined intervention group outperforming either the cognitive-only or physical-only therapy groups. It can be explained by the timing of the different activities in the combined group. Evidence suggests that the timing of physical and mental activities in combined interventions is crucial, and long delays (even performing tasks on separate days) weaken the positive impact on cognition [25]. In future research, combined interventions should include both physical and cognitive exercises on the same day, instead of the seven-day delay we investigated in this study.

Previous studies on laboratory dogs have found that behavioural enrichment (cognitive, physical, and environmental) has a positive impact on dogs' visuospatial memory, discrimination, and reversal learning ability [17–19]. We have found no effect of intervention therapy on companion dogs' visuospatial memory, and we could not test its impact on dogs' learning ability. The Discrimination and reversal learning were excluded before the analysis due to low repeatability. In the previously mentioned studies, laboratory dogs had to discriminate between sizes and colours (black/white), while in our experiment, the two conditions were location and size combined with colour. Since learning to discriminate between locations and learning to discriminate between characteristics (such as size and colour) are different processes [50], it is understandable why good performance in one mode of learning does not necessarily indicate good performance in the other. Because the Discrimination and reversal learning test was not repeatable, there is no point in analysing the difference between the two test occasions, as it does not provide any meaningful insight into the individual's change of capabilities.

Our intervention therapies also failed to influence dogs' manipulative persistency, problem solving ability, and propensity to learn to form eye contact with humans. It could be possibly explained by the physically and cognitively stimulating and challenging environment of companion dogs. They often receive various forms of training, which could potentially hinder the effectiveness of interventional therapies. The dogs' performance on all the previously mentioned tasks was reported to be associated with their training level [28, 51–53]. Future studies should incorporate significantly more challenging tasks for companion dogs than those used with laboratory dogs.

### Impact of other moderators

#### Age

Less old dogs became more sociable and friendlier with the stranger during the Greeting test on their second test occasion. Previous research has already reported that older dogs tend to be less friendly and less social, both with other dogs and with humans [54–57]. However, other studies did not find an association between sociability and age [58, 59]. The reduced sociability can be considered a part of natural age-related decline. While it may be reversible in younger dogs, it's possible that after a certain age, this decline becomes irreversible, and interventions may prove ineffective.

Furthermore, less old dogs were more likely to improve their performance during the Problem solving test and manipulate the new toy during the Novel object recognition. The problem solving ability and neophilia of dogs were also found to be affected by age [59–63]. Younger dogs were more successful in problem solving, while older dogs showed higher levels of neophobia.

Interventions were reported to be more effective when applied to younger individuals [22, 64, 65], that is, when started at an earlier age. Younger individuals may have higher cognitive plasticity, making them more responsive to these interventions [3]. It is important to note that the median age of dogs in our study was 10.45 years, and the youngest dog in our sample was 7.68 years old. This suggests that even relatively old dogs can experience improvements, and it appears that dogs aged 8 to 10 years possess sufficient cognitive plasticity to respond positively to both physical and cognitive interventions.

#### Training level

Less trained dogs were more likely to exhibit new behaviours for food rewards during the Clicker game on their second test occasion. This can be due to the common training style, which primarily emphasizes inhibition and perseverance, rather than the offering of new behaviours. Therefore, more trained dogs may be more restricted, often repeating typical obedience tasks for which they have been trained and rewarded countless times. In contrast, less trained dogs may be less fixated on what has worked for them over the years when offering behaviours.

The literature on the impact of lifelong training on dogs' cognitive decline is controversial. Some studies have found that it can counteract age-related cognitive decline [27, 28], while others have reported no effect of lifelong training [16, 29]. Future studies should investigate which aspects of life-long training can counteract of age-related decline and which cognitive abilities the training beneficially affects.

#### Health status

Healthier dogs were less likely to manipulate the new toy during the Novel object recognition on their second test occasion. The Preference for novelty component showed a moderate positive association with the common cognitive factor in dogs, which age-related decline was more pronounced in individuals with worse health status [36]. Presumably, healthier dogs had a higher baseline performance, potentially leaving less room for improvement. Future studies should examine the relationship between health status and age-related decline in dogs.

#### Play with the owner

The beneficial effect of play on cognitive development has been investigated thoroughly in the past [66–70], and it seems to be a promising tool for treating dementia [71–73]. It is plausible that play also has similar functions in dogs [74].

We found that dogs that played with their owner for more than an hour on a daily basis were more likely to manipulate the new toy during the Novel object recognition on their second test occasion. Since we found no association between play with the owner and manipulating the familiar toy during the Novel object recognition, it suggests that it may have affected dogs' cognitive abilities, not just their playfulness. This includes their attention to detail, information processing, and their inclination toward novelty. Based on our findings, we cannot definitively conclude that play protects against age-related cognitive decline in dogs, but it would be worthwhile to conduct further in-depth studies on this topic. We recommend that future studies investigate the effects of play intervention on dogs' cognitive decline, considering different types of play (e.g., individual, intraspecies, interspecies).

#### Activity/excitability personality trait

Active/excitable dogs became friendlier with the stranger during the Greeting test. Furthermore, they were more likely to exhibit new behaviours for food rewards during the Clicker game on their second test occasion. Their performance was more stable in relation to the exhibition of old behaviours, but if their performance changed, it was more likely to improve than to decline. These results suggest that dogs' personality can also affect their behaviour change over time. However, since personality changes across a dog's lifespan [59], the connection between behaviour change and personality change would be an interesting phenomenon to investigate further in the future.

### Limitations

In our study, we observed negative associations with the baseline score. In other words, dogs with lower baseline performance scores improved, while those with higher scores performed worse across all tests, except for one variable. This pattern is likely the result of a statistical concept known as regression to the mean [75, 76], which means that extremely high or low initial measurements will tend to move closer to the mean in subsequent sampling. Regression to the mean often occurs when measurements are repeatedly taken on the same subject. This happens because values are observed with a non-systematic variation around a true mean [77]. Future studies could potentially address this issue by incorporating two or more baseline measurements or using other statistical means to control for intra-individual variability before implementing intervention therapies and conducting an equal number of measurements after the therapies to overcome the influence of regression to the mean. By using two or more measurements, we can obtain a better estimate of each subject's true mean before and after any intervention [77].

Other limitations were the small sample size and the use of a convenience sample comprising diverse dogs. Therefore, we were unable to balance our sample with respect to life experiences. Nevertheless, we believe that our study provides a basis for future research to investigate further the impact of training levels, owner attitudes or health status on the success of intervention therapies.

We also pre-screened dogs to ensure they were free from serious mobility issues, visual impairments, and hearing impairments. Additionally, we indirectly assessed the owners because they needed to visit our department frequently. As a result, our sample probably consisted mostly of enthusiastic owners who may engage more with their dogs compared to the average population, as well as well-socialized dogs in relatively good physical condition. This likely led to another challenging issue: the ceiling effect [78]. Tasks may have been too easy for these dogs, making it difficult to measure improvement caused by intervention therapy, as their high baseline performance might have left little room for improvement. A more accurate measurement of improvement might be possible in dogs that start from a worse stage. To address this, future studies should include less successfully aging dogs and/or longer intervention therapies, accompanied by more challenging tasks.

In summary, our results indicate that interventional therapies can have an impact on the behaviour of old, healthy pet dogs. Less old dogs, around eight years old, appear to benefit more from these therapies, suggesting their greater effectiveness when initiated at a younger age, as age-related decline may become irreversible beyond a certain point, or sensitivity to intervention therapies may decrease in older age.

## Statements and Declarations

The authors declare no conflict of interest.

## Supplementary Information

Below is the link to the electronic supplementary material.

Supplementary file1 (PDF 303 KB)

Supplementary file2 (XLSX 163 KB)

## Data Availability

The data used for the analyses are provided as Supplementary material.
