# Supplementary material for: The behavioural effect of short-term cognitive and physical intervention therapies in old dogs
Source: GeroScience. 2024 Apr 3;46(6):5409–29. doi: 10.1007/s11357-024-01122-2 (PMC11493909; doi:10.1007/s11357-024-01122-2)
Supplement: Supplementary file 1 — Supplementary file1 (PDF 303 KB) [file 11357_2024_1122_MOESM1_ESM.pdf]

Supplementary Material

Title: The behavioural effect of short-term cognitive and physical intervention therapies in old dogs

Zsófia Bognár [1,2,3]*† (ORCID: 0000-0002-5308-3394), Dóra Szabó[2,3]† (ORCID: 0000-0001-7902-7713), Borbála Turcsán[1,2,3] (ORCID: 0000-0002-0197-5243), Enikő Kubinyi [1,2,3,4] (ORCID: 0000-0002-4468-9845)

[1] MTA-ELTE Lendület "Momentum" Companion Animal Research Group, Budapest, 1117, Hungary
[2] Department of Ethology, ELTE Eötvös Loránd University, Budapest, 1117, Hungary
[3] Senior Family Dog Project
[4] ELTE NAP Canine Brain Research Group

*Corresponding author. *E-mail address:* zsofia.bognar@ttk.elte.hu, *Postal address*: Eötvös Loránd University, Pázmány Péter sétány 1/c, 1117, Budapest, Hungary

†These authors contributed equally to this work

Detailed protocol

The test battery took place in two experimental rooms on two days. Tasks 1-3, 6-9, and 11 were conducted in Room 1 (measured 5 x 6 m), tasks 4-5, 10, and 12 were done in Room 2 (measured 3 x 5 m). Tasks 1-11 were conducted on the first day and task 12 was carried out 1 to 70 days after the other tasks (median: 9 days). The procedure and the rank order of the tasks were the same in both test occasions, but the objects used in the Exploration test, the toys used in the Novel object test and the plates used in the Learning test were different.

Task 1: Exploration test [1]

The room included 16 objects to explore. A chair was placed next to the wall, and four larger objects placed in the corners (a large cardboard box with a pink plastic bowl filled with plastic bags placed on top; a small table with a basket filled with plastic bags placed on top; a bedsheet with a small cardboard box on top filled with shredded paper; a waste bin filled with shredded paper) were present in all setups. The other 11 objects were selected from a pool of small-sized everyday objects with different colours and materials. These were placed in a circle around the middle of the room and along the walls.

The owner and the dog on a leash entered the room together. In the first (*Leashed*) phase of the test, the owner stood for 20 seconds near the closed door without interacting with the leashed dog. In the second (*Unleashed*) phase, the owner took off the leash on the experimenter's signal from outside and released the dog, ignoring the dog after that. For 120 seconds, the dog was free to explore the room while the owner remained standing next to the closed door. If necessary, the owner could give a release command to the dog at the beginning of this test.

Task 2: Box rustle test [1]

The owner moved slowly around the room, searching for four metal coins, one hidden in each of the four containers placed in the corners of the room. The owner was instructed to visit the locations in a fixed order (from right to left), spend at least 10 seconds at each location, and ignore the dog. The dog was free to move around. The total duration of this task was 60 seconds, and the experimenter's signal from outside marked the end.

Task 3: Greeting test [2, 3]

In the first (*Greeting*) phase, the owner leashed the dog and went to the middle of the room. The experimenter entered the room, approached the dog-owner pair (stopping ~ 1 m from the owner), greeted the owner, and collected the coins retrieved during the Box rustle test. Then the experimenter turned towards the dog and greeted it. If the dog showed friendly (stepping towards the experimenter, tail wagging) or neutral (no overt signs of friendliness, fear, or aggression) behaviour, the experimenter petted the dog while continuously speaking to the dog in a friendly way. Then the experimenter stepped 1 m sideways within the reach of the leash. If the dog followed, the experimenter petted the dog again. If the dog did not follow, the experimenter called again and crouched but did not make contact unless the dog initiated it. If the dog initially avoided the experimenter or showed fearful behaviour (without barking, growling, or any aggressive

display), the experimenter crouched down and called the dog. If the dog approached the experimenter in a non-aggressive manner, the experimenter followed the procedure described above. If the dog did not respond, the experimenter did not try to make direct contact with the dog; instead, she ignored the dog and talked to the owner for 30 sec. If the dog initially growled or barked at the experimenter, she remained out of reach of the leash and talked continuously to the dog in a friendly manner for 10 sec while avoiding eye contact and then terminated the test (there were no such cases while testing the current sample of family dogs).

In the second phase (*Object play*), the experimenter went out of the room and brought in a tug toy and a ball. The experimenter tried to engage the dog in playing with the toys for 30 sec (tug-of-war game or ball play, depending on which toy the dog was more interested in).

Task 4: Pointing test [4]

In the first phase (*Warm-up trial*), we aimed to ensure that the dog was comfortable approaching and eating from the containers (pots) used in the test trials. Upon entering the room, the owner sat down on the chair at the starting position (3 m from the experimenter's position), took off the leash, and held the dog by the collar. The experimenter called the dog's attention by saying, "dog name + look", showed the treat, then dropped it into the pot and put it in front of her on the ground. Then the dog was released and allowed to take the food. The owner and the experimenter were allowed to encourage the dog if necessary.

In the second phase (*Test trials*), the owner was sitting in his/her chair holding the dog by the collar at the starting position, and the experimenter standing 3 m from them, holding two identical pots folded into each other. The experimenter called the dog's attention ("dog name + look"), and after establishing eye contact, she showed the treat and dropped it into the upper pot. She then shuffled the two pots 2-3 times and placed them on the marks on the floor to her left and right (the distance between the two pots was 1.5 m). She then called the dog's attention again and performed a momentary distal pointing gesture (3 seconds) to the baited pot. After the experimenter returned to her starting position (both hands held in front of her chest), the owner released the dog. After the dog made a choice (its nose came within 10 cm of the pot), the experimenter removed the other pot before the dog had the chance to investigate it. If the dog made a correct choice, it was allowed to eat the food. If the dog made an incorrect choice, the baited pot was only shown to the dog. At the end of the trial, the owner called or led the dog back to the start position, and the next trial began. There were six test trials, with the first three trials on the same side and the second three on the other side. The location of the first baited pot was counterbalanced among dogs.

Task 5: Manipulative persistency test [5]

In the first phase *(Solvable trial)*, the experimenter showed the toy to the dog (Kong Wobbler^{TM}, small or large, depending on the dog's size), then baited the toy in front of the dog with 20 pieces of small-sized treats, which could be retrieved by manipulating the toy. The toy was then placed in the middle of the room, and the dog had 60 seconds to manipulate it while the experimenter and the owner remained in the same positions as in the previous test. If the dog lost interest, the owner was allowed to encourage verbally and via pointing at the toy without leaving his/her chair.

In the second phase *(Unsolvable trial),* the test was repeated with the same procedure, but this time, the experimenter baited the toy with a large treat that the dog could not obtain.

After this test, there was a break (5-10 minutes) during which the dog remained outside of the test rooms. Then, room 1 was rearranged, removing all the objects.

Task 6: Clicker game [6]

The owner sat on the chair next to the door and was asked not to communicate or interact with the dog during the test. The experimenter stood in the centre of the room with a food pouch filled with sausages on her belt and holding a sound-making device (similar to a clicker but displaying a different sound). She called the dog to her, then asked it to sit. Once the dog sat in front of her, she clicked and threw a piece of sausage on the floor. After that, the experimenter remained motionless but clicked and rewarded the dog for presenting any novel behaviour, both object- and body-related. If the dog presented the same behaviour repeatedly, the experimenter waited until a new behaviour was offered while also encouraging the dog by smiling and nodding but without speaking. The test lasted for two minutes, measured from the first click sound.

Task 7: Problem solving test [7]

The apparatus had the following dimensions: a 62.5 cm x 53 cm platform with a 22.5 cm x 22.5 cm x 38 cm rectangle box (opaque or transparent) attached to it. The box was closed on the top, bottom, and three sides, with only one side left open.

In all trials, the owner sat on a chair 1.5 m from the apparatus, held the dog by the collar until the experimenter baited the apparatus and returned to her starting position next to the owner. Then the owner let the dog free, and the dog had 45 seconds to obtain the reward. The owner was allowed to encourage the dog verbally and via pointing at the apparatus without leaving his/her chair. If the dog did not succeed in a given trial, the experimenter provided the minimal necessary help to the dog to get the reward to prevent loss of motivation. In the first phase *(Opaque, trials 1-3),* the apparatus was opaque (wood), the dog could see the baiting process, and the opening was always in the middle position, facing away from the dog.

In the second phase *(Transparent, trials 4-10),* the apparatus was transparent (plexiglass) (so the dog was able to see the food reward inside). In these trials, the experimenter prevented the dog from seeing the baiting process (i.e., the location of the opening on the apparatus) via a visual barrier. The location of the opening was on the same side (left or right) in trials 4 to 6, shifted to the opposite side (right or left) in trials 7 to 9, then shifted to the middle position (facing away from the dog) in trial 10. The location of the opening in trial 4 was counterbalanced among subjects.

Task 8: Attention test [8, 9]

The owner sat on a chair approximately 4.5 m from the wall where the stimuli were presented. The owner was told to ignore both the dog and the actions of the experimenter. The dog was positioned next to the owner at the beginning of each context and remained leashed during the entire test. In the *Non-social trial* (flying object), the experimenter remotely manipulated a yellow plastic frisbee

from outside the room by pulling a fishing line through a metal loop in the ceiling in the testing room. The object moved up and down (seemingly on its own) next to the wall facing the dog for one minute. In the *Social trial* (‘painting’ the wall), the experimenter entered the testing room, silently walked to the wall, and, with her back to the dog, made up-down movements (as if painting the wall). After one minute, the experimenter left the room without looking at the dog.

Task 9: Training for eye contact [3, 6, 8]

In the first phase *(Training),* the dog was unleashed, the owner sat on a chair next to the door and was told to ignore the dog. The experimenter stood in the center of the room holding a sound-making device in one hand. Both hands were positioned in a relaxed posture by her sides. The experimenter had a food pouch on her belt, positioned at her back. First, the experimenter called the dog's attention and threw a piece of food on the floor. Then she remained motionless, and whenever the dog established eye contact with her, the experimenter clicked and tossed a piece of food on the floor. This phase lasted for 20 eye contacts or a maximum of 5 minutes.

There were two conditions in the second phase (*Sustained eye contact*): silent and with distraction, and the order was counterbalanced among dogs. In both conditions, the dog had to keep eye contact with the experimenter for gradually increasing durations to get rewarded. Each condition had five levels: 2 sec, 5 sec, 10 sec, 20 sec, 40 sec. Once the dog has successfully passed a level, the latency between establishing eye contact with the experimenter and the click + reward was increased to the next level. The dog had three attempts to pass each level. If the dog failed all three attempts, the test was terminated. In the "with distraction" condition, white noise was played in the background (the mean level of the sound was 49 dB). The dog received three eye contact retraining trials between the two conditions to maintain motivation.

Task 10: Novel object recognition [10]

The toys used in the test were selected from a pool of six dog toys, and their combination was counterbalanced between dogs. In the first phase (*Passive familiarization*), the experimenter, the owner, and the dog entered the room, the owner sat down on a chair, let the dog free, and afterwards ignored the dog. There were two identical dog toys on the floor, 2 m apart from each other and 1.2 m from the dog. The dog was free to interact with them for 30 seconds.

In the second phase (*Active familiarization*), the experimenter approached and interacted with both toys (picked them up individually and engaged the dog's attention by saying, "what do I have" and "look at this" in a happy voice). This phase also lasted for 30 seconds. After phase 2, the owner and the dog left the room for 5 minutes while the experimenter switched one of the toys to a new one.

In the third phase (*Test phase*), after re-entering the room, the owner sat down, released the dog, and the dog was free to interact with the toys for 60 seconds.

Task 11: Memory test [11]

There were five identical pots on the floor, positioned at an equal distance (3 m) from the starting position of the dog, each pot 1.6 m from the other in a semi-circular arrangement. The

experimenter, the owner, and the dog entered the room, the owner and the dog walked to the starting position. The experimenter called the dog's attention, showed a piece of food, walked to a pre-selected pot in a straight line from the start, and put the reward in the pot. Then the experimenter, the owner, and the dog left the room, and outside the owner distracted the dog by giving simple commands or petting and talking to the dog. After 30 seconds, the experimenter, the owner, and the dog re-entered the room, went to the starting position, and the owner released the dog. The trial ended when the dog found the treat. There were five trials in total, and each container was baited once in a pre-defined order. The order of the baited locations was counterbalanced across subjects.

Task 12: Discrimination and reversal learning [12–14]

The task used two stimulus types. In the *location* type, the stimulus was a blue plastic plate (flat, round, 20 cm in diameter), and the discrimination was based on the location of this plate (left or the right-hand side of the experimenter). In the *colour* type, the stimuli were two plates: a white plastic plate (deep, round, 12 cm in diameter) and a black plastic plate (flat, rectangular, length: 23 cm, width: 15.5 cm). The dogs received one type in their baseline measurement and the other type in their second test occasion.

The task contained two phases, a discrimination phase and a reversal phase. In both phases the dogs received positive (P) and negative stimuli (N) in 50 consecutive trials, which were presented in a fixed semi-random order (PPNPNN, repeated until the criteria (see below) was reached or for a maximum of 50 trials). The positive plate always contained a small piece of food, while the negative plate was always empty. The side and colour that was used as the positive stimulus were counterbalanced among the dogs.

At the beginning of each trial, the owner was sitting on a chair approximately 3 m from the experimenter and held the dog by the collar or leash. The experimenter turned its back to the owner and the dog and baited (or pretended to bait) a plate. Then she turned back and called the dog's attention ("name + look"). Once she established eye contact with the dog, she put the plate on its pre-determined location (left or right side, ~1m from the experimenter in the location type, or in front of the experimenter, halfway between the left and right locations in the colour type). The owner was instructed to let the dog go immediately as the plate touched the floor. If the dog did not start moving when released, the owner was allowed to encourage it verbally (e.g., "Go!", "It's yours") or by gently touching it. Apart from this, no other forms of communication were allowed. The dog had 15 seconds to reach the plate (and, in the case of P, eat the food), then the experimenter picked up the plate, and the owner called the dog back for the subsequent trial.

In each trial, the experimenter recorded the latency to reach the plate, measured from the moment the plate touched the floor until the dog was <15 cm of the plate (defined by markings on the floor). If the dog did not approach the plate, the experimenter gave the maximum latency (15 sec). The dog was deemed to have learned the association between the stimulus and the food when the longest latency in the last five positive (P) trials was shorter than any latency in the last five negative (N) trials. The testing was terminated if the dog did not reach this criterion within 50 trials or refused to leave the chair's proximity for three consecutive trials. If the dog passed the criteria

of the discrimination phase, the test continued with the reversal phase after a short break. In the reversal phase, the procedure was the same, but the P and N locations or colours were switched, i.e., if the P was on the left side, it became the right side. Again, the dog had a maximum of 50 trials to learn the reversed association, the criteria of learning was the same as in the discrimination phase.

Exclusion of coded variables

We used a top-down approach and coded/scored a broad list of behavioural measures. The list was then reduced in three steps. We initially coded 100 variables, based on what was coded in the previous studies related to our tasks, as well as our preliminary observations (pilot tests). From this list, we excluded variables (N = 25) with low variability and/or with high skewness (caused by a few outlier values). Such variables are either not suited to detect individual differences in the performance, or not sensitive enough therefore differentiate only between the extremes. The criteria for exclusion were determined for each variable type separately. For durations (all with the hypothetical range of 0% to 100%), a variable was rejected if its range was < 50, and/or its mean was < 10. For latencies and frequencies, we excluded variables in which more than 50% of the dogs received the same value (mostly 0 or maximum). For nominal scores (all with four hypothetical discrete values: 0 to 3), a variable was excluded when more than 70% of the dogs received the same value or when a variable had a bimodal distribution (i.e., less than 10% of the dogs received the two least frequent values combined). We also removed redundant variables (14 variables), that is, when the same behaviour was measured with multiple different types of scales (e.g., latency, frequency, and duration), because these variables are closely (mechanistically) linked to each other which would bias the analyses. The remaining 61 variables were used in the analyses.

Table S1. Definition and inter-observer reliability (assessed by intraclass correlation (ICC)) of the variables coded in the twelve tasks. E: experimenter, O: owner

**Task/Phase**	**Variable**	**Definition**	**ICC**	**p**
**Task 1: Exploration test [1]**				
**on leash**	Time% activity	Moving the legs: 0: nearly 0%; 1: <50%; 2: =>50%; 3: nearly 100% of the time	0.71	<0.001
**free**	Time% activity	Moving the legs: 0: nearly 0%; 1: <50%; 2: =>50%; 3: nearly 100% of the time	0.87	<0.001
**free**	Time% proximity to O	being < 1 m to O: 0: nearly 0%; 1: <50%; 2: =>50%; 3: nearly 100% of the time	0.75	<0.001
**free**	N of objects visited	nose < 10 cm from object: 0: 0-1 objects; 1: 2-5 objects; 2: 6-10 objects; 3: 11-16 objects	0.97	<0.001
**Task 2: Box rustle test [1]**				
	Search box, 1st to 3rd	When O investigates the box, the dog 0: does not look; 1: looks but no approach; 2: approaches <1m but no touch; 3: touches the box	0.75-0.88	<0.001 for all
	Time% following O	When O walks among the boxes, the dog follows O: 0: nearly 0%; 1: <50%; 2: =>50%; 3: nearly 100% of the time	0.77	<0.001
**Task 3: Greeting test [2, 3]**				
**greeting**	Approach E	When the E greets the dog, the dog approaches her: 0: never; 1: when crouching; 2: when calling; 3: when standing	0.88	<0.001
**greeting**	Follow E	When the E steps away, the dog approaches her: 0: never; 1: when crouching; 2: when calling; 3: when standing	0.96	<0.001
**greeting**	Time% tail-wagging	During the greeting, the dog wags its tail: 0: nearly 0%; 1: <50%; 2: =>50%; 3: nearly 100% of the time	0.97	<0.001
**greeting**	Intensity tail-wagging	During the greeting, the maximum amplitude of tail-wagging: 0: no tail wagging; 1: 0-45 degree; 2: 46-90 degree; 3: >90 degree	0.89	<0.001
**object play**	Play with E	When the E initiates play with the dog, the dog 0: does not play; 1: mouths/touches the toy a bit; 2: plays with little enthusiasm; 3: plays enthusiastically most of the time	0.96	<0.001
**Task 4: Pointing test [4]**				
	N of correct choices	Frequency of times the dog chooses the pot with food (out of 6)	1.00	<0.001
**Task 5: Manipulative persistency test [5]**				
**solvable**	Time% look at the Kong	Duration of looking at the Kong	0.99	<0.001
**solvable**	Time% touch the Kong	Duration of manipulating (touching, pawing, mouthing, nosing) the Kong	0.96	<0.001
**unsolvable**	Time% look at the Kong	Duration of looking at the Kong	0.99	<0.001
**unsolvable**	Time% touch the Kong	Duration of manipulating (touching, pawing, mouthing, nosing) the Kong	0.99	<0.001

Task/Phase	Variable	Definition	ICC	p
**Task 6: Clicker game [6]**				
	N of repeated behaviors	Frequency of previously rewarded behaviors repeated (except looking at E)	0.76	<0.001
	N of novel behaviors	Frequency of new behaviors	0.98	<0.001
	Time% repeated behaviors	Duration of performing previously rewarded behaviors (except looking at E)	0.97	<0.001
	Time% passivity	Duration of staying still (sitting, lying, standing in one place)	0.96	<0.001
	N of food-related behaviors	Frequency of behaviors aimed at obtaining the food (touching E's hand, the clicker, or the food bag, circling or jumping at E)	0.42	0.028
	N of obedience tricks	Frequency of previously learned tricks performed, e.g., sit, lay down, giving paw, turning around, etc.	0.89	<0.001
**Task 7: Problem solving test [7]**				
**opaque**	Latency to find food, 1st to 3rd trials	From the moment the dog starts moving until the dog reaches into the box (or maximum: 30sec)	0.69-0.92	<0.001 for all
**transparent**	Latency to find food, 4th to 10th trials	From the moment the dog starts moving until the dog reaches into the box (or maximum: 30sec)	0.82-0.99	<0.001 for all
**all trials**	N of correct first choices	Frequency of times the dog finds the food in the first choice (out of 10)	0.70	<0.001
**Task 8: Attention test [8, 9]**				
**non-social**	Latency to look away object	From the moment the frisbee starts moving until the dog looks away: 0: < 5 sec; 1: 5 - < 16 sec; 2: 16 - < 30 sec; 3: => 30 sec	0.92	<0.001
**non-social**	Time% look at the object	0: nearly 0%; 1:<50%; 2: =>50%; 3: nearly 100% of the time	0.61	0.002
**social**	Latency to look away human	From the moment the door opens until the dog looks away: 0: < 5 sec; 1: 5 - < 16 sec; 2: 16 - < 30 sec; 3: => 30 sec	0.95	<0.001
**Task 9: Training for eye contact [3, 6, 8]**				
**training**	Mean eye contact latency	Mean latency of the first 15 eye contacts (from the moment the dog takes the sausage into its mouth until E clicks) (or maximum: 60 sec)	1.00	<0.001
**training**	Latency to learn	If the dog passed the training criteria, the sum of the first 15 eye contact's latency; if the dog did not pass the training criteria: 300 sec	1.00	<0.001
**sustained**	Sustained eye contact	Maximum duration of sustained eye contact: 0: did not pass first level; 1: 2 sec; 2: 5 sec; 3: 10 sec; 4: 20 sec; 5: 40 sec. Mean of the with and without distraction conditions	0.64	<0.001

Task/Phase	Variable	Definition	ICC	p
**Task 10: Novel object recognition [10]**				
**passive familiarization**	N of approach any toys	Frequency of approaching any of the toys <20cm	0.69	<0.001
**passive familiarization**	N of touch any toys	Frequency of touching any of the toys	0.83	<0.001
**passive familiarization**	Time% proximity to any toys	Duration of being <20cm to any of the toys	0.86	<0.001
**passive familiarization**	Time% touch any toys	Duration of touching any of the toys	0.81	<0.001
**test phase**	N of approach familiar toy	Frequency of approaching the familiar toy <20cm	0.65	<0.001
**test phase**	N of approach new toy	Frequency of approaching the new toy <20cm	0.49	0.014
**test phase**	Time% proximity to familiar toy	Duration of being <20cm to the familiar toy	0.93	<0.001
**test phase**	Time% proximity to new toy	Duration of being <20cm to the new toy	0.93	<0.001
**test phase**	N of touch familiar toy	Frequency of touching the familiar toy	0.53	0.008
**test phase**	N of touch new toy	Frequency of touching the new toy	0.49	0.014
**test phase**	Time% touch familiar toy	Duration of touching the familiar toy	0.85	<0.001
**test phase**	Time% touch new toy	Duration of touching the new toy	0.92	<0.001
**Task 11: Memory test [11]**				
	Latency to find food, 1st to 5th trials	From the moment the dog starts moving until the dog's nose enters into the pot (or maximum: 30sec)	0.83-1.00	<0.001 for all
	N of correct first choices	Frequency of times the dog finds the food in the first choice (out of 5)	1.00	<0.001
**Task 12: Discrimination and reversal learning [12–14]**				
**discrimination**	Number of learning trials	The number of trials required to learn the initial association between the stimuli and reward until criteria; if the dog did not pass training criteria: 50.	0.74	<0.001
**reversal**	Number of reversal trials	The number of trials required to learn the reversed association between the stimuli and reward; if the dog did not pass training criteria: 50.	0.86	<0.001

Table S2. Description, internal consistency and task reliability assessments of the cognitive measures. A PCA was run on the coded variables for ten tasks (all, except the Pointing and Learning test). The task reliability has been assessed using intraclass correlation (ICC). O: owner, E: experimenter

Task/Phase	Variable	Component 1	Component 2
**Task 1: Exploration test**		*Activity*	
on leash	Time% activity	**0.500**	
free	Time% activity	**0.914**	
free	Time% proximity to O	**-0.845**	
free	N of objects visited	**0.864**	
Explained variance (%)		*63.655*	
Cronbach's alpha		*0.756*	
ICC		*0.348, p=0.040*	
**Task 2: Box rustle test**		*Following*	
	Search box, 1st	**0.752**	
	Search box, 2nd	**0.718**	
	Search box, 3rd	**0.765**	
	Time% following O	**0.717**	
Explained variance (%)		*54.499*	
Cronbach's alpha		*0.649*	
ICC		*0.454, p=0.007*	
**Task 3: Greeting test**		*Friendliness*	
greeting	Approach E	**0.833**	
greeting	Follow E	**0.772**	
greeting	Time% tail-wagging	**0.894**	
greeting	Intensity tail-wagging	**0.882**	
object play	Play with E	**0.686**	
Explained variance (%)		*66.758*	
Cronbach's alpha		*0.837*	
ICC		*0.874, p<0.001*	
**Task 4: Pointing test**		*Following pointing gesture*	
	Number of correct choices	*No PCA*	
ICC		*-0,088, p = 0.637*	
**Task 5: Manipulative persistency test**		*Persistency*	
solvable	Time% look at the Kong	**0.914**	
solvable	Time% touch the Kong	**0.907**	
unsolvable	Time% look at the Kong	**0.916**	
unsolvable	Time% touch the Kong	**0.915**	
Explained variance (%)		*83.336*	
Cronbach's alpha		*0.931*	
ICC		*0.836, p<0.001*	

**Task/Phase**	**Variable**	**Component 1**	**Component 2**
**Task 6: Clicker game**		*Flexibility*	*One-trial learning*
	N of repeated behaviours	0.371	**0.811**
	N of novel behaviors	**0.646**	0.378
	Time% repeated behaviors	**-0.584**	**0.668**
	Time% passivity	**-0.850**	0.006
	N of food-related behaviors	**0.706**	0.008
	N of obedience tricks	0.058	**0.921**
Explained variance (%)		*42.865*	*28.148*
Cronbach's alpha		*0.645*	*0. 728*
ICC		*0.550, p<0.001*	*0.767, p<0.001*
**Task 7: Problem solving test**		*Problem-solving success*	
opaque	Latency of finding the food, 1st trial	**-0.558**	
opaque	Latency of finding the food, 2nd trial	**-0.782**	
opaque	Latency of finding the food, 3rd trial	**-0.768**	
transp.	Latency of finding the food, 4th trial	**-0.764**	
transp.	Latency of finding the food, 5th trial	**-0.831**	
transp.	Latency of finding the food, 6th trial	**-0.794**	
transp.	Latency of finding the food, 7th trial	**-0.835**	
transp.	Latency of finding the food, 8th trial	**-0.834**	
transp.	Latency of finding the food, 9th trial	**-0.818**	
transp.	Latency of finding the food, 10th trial	**-0.705**	
all trials	Number of correct first choices	does not load >0.5	
Explained variance (%)		*59.771*	
Cronbach's alpha		*0.923*	
ICC		*0.531, p<0.001*	
**Task 8: Attention test**		*Attention to Object*	
non-social	Latency to look away object	**0.928**	
non-social	Time% look at the object	**0.928**	
social	Latency to look away human	does not load >0.5	
Explained variance (%)		*86.050*	
Cronbach's alpha		*0.817*	
ICC		*0.315, p=0.059*	
**Task 9: Training for eye contact**		*Associative learning*	
training	Mean eye contact latency	**-0.878**	
training	Latency to learn	**-0.936**	
sustained	Sustained eye contact	**0.848**	
Explained variance (%)		*78.852*	
Cronbach's alpha		*0.865*	
ICC		*0.866, p<0.001*	

Task/Phase	Variable	Component 1	Component 2
**Task 10: Novel object recognition**		*Preference for familiar*	*Preference for novelty*
passive familiarization	N of approach any toys	0.126	**0.622**
passive familiarization	N of touch any toys	0.211	**0.617**
passive familiarization	Time% proximity to any toys	**0.746**	0.266
passive familiarization	Time% touch any toys	**0.769**	0.193
test phase	N of approach familiar toy	**0.565**	0.326
test phase	N of approach new toy	0.155	**0.691**
test phase	Time% proximity to familiar toy	**0.957**	-0.238
test phase	Time% proximity to new toy	-0.196	**0.890**
test phase	N of touch familiar toy	**0.721**	0.067
test phase	N of touch new toy	0.010	**0.777**
test phase	Time% touch familiar toy	**0.916**	-0.261
test phase	Time% touch new toy	-0.188	**0.869**
Explained variance (%)		*39.833*	*24.203*
Cronbach's alpha		*0.886*	*0.848*
ICC		*0.709, p<0.001*	*0.621, p<0.001*
**Task 11: Memory test**		*Memory*	
	Latency of finding the food, 1st trial	**-0.598**	
	Latency of finding the food, 2nd trial	**-0.632**	
	Latency of finding the food, 3rd trial	**-0.637**	
	Latency of finding the food, 4th trial	**-0.686**	
	Latency of finding the food, 5th trial	**-0.540**	
	Number of correct first choices	**0.823**	
Explained variance (%)		*43.384*	
Cronbach's alpha		*0.732*	
ICC		*0.677, p<0.001*	
**Task 12: Discrimination and reversal learning**		*Discrimination learning*	*Reversal learning*
	Number of learning trials	No PCA	No PCA
ICC		*-0,147, p=0.716*	*0.242, p = 0.124*

References

1. Turcsán B, Wallis L, Virányi Z, et al (2018) Personality traits in companion dogs—Results from the VIDOPET. PLoS One 13:e0195448. https://doi.org/10.1371/journal.pone.0195448

2. Svartberg K, Forkman B (2002) Personality traits in the domestic dog (Canis familiaris). Appl Anim Behav Sci. https://doi.org/10.1016/S0168-1591(02)00121-1

3. Bognár Z, Szabó D, Deés A, Kubinyi E (2021) Shorter headed dogs, visually cooperative breeds, younger and playful dogs form eye contact faster with an unfamiliar human. Sci Rep 11:9293. https://doi.org/10.1038/s41598-021-88702-w

4. Soproni K, Miklósi Á, Topál J, Csányi V (2001) Comprehension of human communicative signs in pet dogs (Canis familiaris). J Comp Psychol 115:122–126. https://doi.org/10.1037/0735-7036.115.2.122

5. Range F, Heucke SL, Gruber C, et al (2009) The effect of ostensive cues on dogs' performance in a manipulative social learning task. Appl Anim Behav Sci 120:170–178. https://doi.org/10.1016/j.applanim.2009.05.012

6. Wallis LJ, Range F, Müller CA, et al (2015) Training for eye contact modulates gaze following in dogs. Anim Behav 106:27–35. https://doi.org/10.1016/j.anbehav.2015.04.020

7. Gerencsér L, Bunford N, Moesta A, Miklósi Á (2018) Development and validation of the Canine Reward Responsiveness Scale –Examining individual differences in reward responsiveness of the domestic dog. Sci Rep 8:4421. https://doi.org/10.1038/s41598-018-22605-1

8. Chapagain D, Virányi Z, Wallis LJ, et al (2017) Aging of Attentiveness in Border Collies and Other Pet Dog Breeds: The Protective Benefits of Lifelong Training. Front Aging Neurosci 9:. https://doi.org/10.3389/fnagi.2017.00100

9. Wallis LJ, Range F, Müller CA, et al (2014) Lifespan development of attentiveness in domestic dogs: drawing parallels with humans. Front Psychol 5:. https://doi.org/10.3389/fpsyg.2014.00071

10. Kaulfuß P, Mills DS (2008) Neophilia in domestic dogs (Canis familiaris) and its implication for studies of dog cognition. Anim Cogn 11:553–556. https://doi.org/10.1007/s10071-007-0128-x

11. Piotti P, Szabó D, Wallis LJ, et al (2017) The effect of age on visuo-spatial short-term memory in family dogs. Pet Behav Sci 17. https://doi.org/10.21071/pbs.v0i4.10130

12. Piotti P, Szabó D, Bognár Z, et al (2018) Effect of age on discrimination learning, reversal learning, and cognitive bias in family dogs. Learn Behav. https://doi.org/10.3758/s13420-018-0357-7

13. Kis A, Hernádi A, Kanizsár O, et al (2015) Oxytocin induces positive expectations about ambivalent stimuli (cognitive bias) in dogs. Horm Behav 69:1–7. https://doi.org/10.1016/j.yhbeh.2014.12.004

14. Mendl M, Brooks J, Basse C, et al (2010) Dogs showing separation-related behaviour exhibit a 'pessimistic' cognitive bias. Curr Biol 20:R839–R840. https://doi.org/10.1016/j.cub.2010.08.030
